# Supplementary material for: Integrating post-validation surveillance of lymphatic filariasis with the WHO STEPwise approach to non-communicable disease risk factor surveillance in Niue, a study protocol
Source: PLoS One. 2025 Jan 16;20(1):e0315625. doi: 10.1371/journal.pone.0315625 (PMC11737670; doi:10.1371/journal.pone.0315625)
Supplement: Attachment — Submitted filename: Response to reviewers_R1.docx [file pone.0315625.s001.docx]

**Response to reviewers’ comments**

**Ref number: PONE-D-24-36104**

**Title: Integrating post-validation surveillance of lymphatic filariasis with the WHO STEPwise approach to non-communicable disease risk factor surveillance in Niue, a study protocol**

| **No.** | **Comment** | **Response** |  |
| --- | --- | --- | --- |
| **Journal requirements** | | | |
| 1 | Please ensure that your manuscript meets PLOS ONE's style requirements, including those for file naming. The | Requirements are met. |  |
| 2 | Please amend your list of authors on the manuscript to ensure that each author is linked to an affiliation. Authors’ affiliations should reflect the institution where the work was done (if authors moved subsequently, you can also list the new affiliation stating “current affiliation:….” as necessary). | Each author has an affiliation. |  |
| 3 | Please include a caption for figure 3a | Figure 4a (note that the number of figures has changed) includes a caption. It is “Fig 4(a). Finger prick capillary puncture” |  |
| 4 | Please include a caption for table 2. | The manuscript does not include Table 2. |  |
| 5 | Please include a complete copy of PLOS’ questionnaire on inclusivity in global research in your revised manuscript. Our policy for research in this area aims to improve transparency in the reporting of research performed outside of researchers’ own country or community. | The questionnaire has been uploaded to the manuscript management system. |  |
| 6 | Please state what role the funders took in the study. | The following text has been added at around line 22. “The funders had no role in study design or decision to publish. They will not be involved in data collection or analysis. The funder had no role in the preparation of this manuscript” |  |
| 7 | Please review your reference list to ensure that it is complete and correct. | The reference list has been reviewed. |  |
| **Reviewer 1’s comments** | | | |
| 1 | I suggest removing hypothesis 3, it feels like a subset of hypothesis 1 | Hypothesis 3 has been removed, as suggested. |  |
| 2 | Line 129 has a pair of errant words | This typo has been corrected. Thank you. |  |
|  | Line 150 were should be "where" | “were” has been replaced with “where” (around line 153). |  |
| 3 | Line 155 please state the age group sampled in the 'C survey' |  |  |
| 4 | Line 164 missing a space in the date | Space added. |  |
| 5 | Line 260 I suggest you use "the house" and not "a house"; I would imagine most people slept in a house (unless you really do mean to distinguish between people sleeping in doors vs. outdoors) | “a” changed to “the” at around line 295. |  |
| 6 | Can you add a little more commentary in the discussion around the applicability of integrating with STEPS surveys? Are they always census surveys in the Pacific? To what other countries would you anticipate this methodology would extend? Several times you mention priority diseases. Please share this list of diseases for Niue to give the reader an idea of what will be assessed, and possibly inspire readers with the types of diseases that can be assessed in an integrated manner. | In response: additional content that:   - provides more details about the approach to integrating PVS for LF with STEPS, including a figure (Fig 3) showing the points in the STEPS workflow at which integration will occur (around line 272) - highlights the WHO recommends STEP surveys be conducted every 3 to 5 years, and hence we expect that we expect the approach outlined to generate interest across the globe (around line 364-369) - list the priority diseases that will be tested has been added around line 224. |  |
| **Reviewer 2’s comments** | | | |
| 1 | Line 76. Ae. Cooki. cooki should be with a small "c". | The typo has been corrected. |  |
| 2 | Line 113: correct the spelling of ESRI ArcGIS | The typo has been corrected. |  |
| 3 | Line 125: Change remined to remained. | The typo has been corrected. |  |
| 4 | Lines 105/128/133/139/149: Check the population number. In the abstract and in line 105, a population of 1600 is reported. In line 128, 4,408 people were tested in 1972. Has the population declined? What accounts for that? | Yes, the population of Niue has declined dramatically. The 2022 census reports an on-island population of 1,681, down from 5,000 in the 1960s. The decrease is primarily attributed to Niue’s free association status with New Zealand. This status allows Niuean citizens to travel to, work, and study in New Zealand without restriction. There are an estimated 27,000 Niueans in Auckland.  To clarify the text around line 130, we have edited it to read “…the survey was reported to have included 98.4% of the population. However, the specifics of the population subset used as the denominator for this calculation are unclear.” |  |
| 5 | Line 129: Remove "nor" after whether. | This typo has been corrected. |  |
| 6 | Line 196: Correct the spelling of "Fourth" | This typo has been corrected. |  |
| 7 | Why has the sampling population been restricted to those aged 15 - 69 years? A clarification for this will be useful. | WHO recommends that the target population for a STEPS NCD risk factor survey be, at a minimum, all adults aged 18 to 69 years (WHO STEPS Manual). Niue DoH decided to include 15-18-year-olds in their STEPS survey. As our PVS work aims to integrate with the STEP survey, we have aligned our sample age.  To explain this, we have edited the text at around line 234. It now reads, “STEPS surveys typically collect data from individuals aged 18 to 69 years. The Niue DoH have decided to extend this to include all people aged 15 to 69 years, and consequently, our study will align.” |  |
| 8 | A description of the STEPS survey and how the LF PVS fits in will be useful. | On review, we feel that the LF PVS integration with STEPs is captured in the section titled ‘Survey implementation’ (around line 260). However, to address the reviewer’s suggestion, we have added the following text and associated figure at around line 272.  “Figure 3 presents a diagram showing at what points LF PVS will integrate with the STEPS survey’s workflow.” |  |
| 9 | Are there other opportunities for integration that could be explored beyond the STEP survey | We expect so; however, discussing other integration options is outside this protocol paper's scope. We will be mindful to include a discussion about the pros and cons and the opportunities and challenges of PVS integration in our results manuscript. |  |
| 10 | Portions of the text described in the "Field Logistics" section should be moved to the "Informed Consent" section. There is some repetitions e.g. consenting procedures. | Text in the ‘field logistics’ section provide an overview while details about how informed consent will be collected are presented in the ‘informed consent’ section. The brief introduction of consent in the field logistics section is needed to maximise clarity. As such, we have not made any major changes. Small edits have been made around lines 266 and 286. |  |
| 11 | Knowing that molecular xenomonioting results in higher infection prevalence in mosquitoes compared to humans, is there any consideration for this in the PVS surveys? | Molecular xenomonitoring is not being considered in Niue at this stage. Also, it is not an obvious fit with the STEPS survey workflow and hence not a good fit for integration. |  |
| 12 | It would be useful to provide copies of the tools used (e.g. questionnaires) as supplementary files to guide the reproducibility of the work by others. | Ideally, we agree. However, the survey tools are still being drafted. We intend to include these in our results manuscript. |  |
| 13 | Some potential benefits of integration were listed. It might also be helpful for the authors to describe any challenges as part of the integration of the LF survey and STEP, even at the planning stage. | In response, at around line 209 we have added “We recognise that challenges may arise, including difficulties in modifying established survey processes to enable integration, reaching a consensus on the most suitable procedures and workflows, and potential conflicts when the prioritisation of field activities benefits one programme over another. We plan to identify and address these challenges through early and open communication and the development of joint implementation plans.” |  |
| **Reviewer 3’s comments** | | | |
| 1 | Abstract: Consider revising the title, specifying the disease for integration with LF. It can be “Integrating post-validation surveillance of lymphatic filariasis with a standard STEPs survey in Niue, a study protocol” | We have amended the title. It now reads “Integrating post-validation surveillance of lymphatic filariasis with the WHO STEPwise approach to non-communicable disease risk factor surveillance in Niue, a study protocol” (line 1-4). |  |
| 2 | Line 24-29: Can be deleted. Start with LF situation in Niue. | We have edited out much of the text the reviewer suggests be removed. |  |
| 3 | Line 32-34: Rephrase the sentence as “However, no surveillance was conducted ever since validation to confirm infection rates have remained below WHO’s elimination threshold”. | The change has been made around line 34. The passage now reads, “ However, no surveillance has been conducted since validation to confirm infection rates have remained below WHO’s elimination threshold.” |  |
| 4 | Line 42: survey can be deleted | The word “survey” has been deleted. |  |
| 5 | Line 47: Consider replacing “for ongoing LF transmission” with “for LF”. Prevalence of antigenemia not necessary indicate the ongoing transmission. It indicates both past and recent exposure. | The word “ongoing” has been deleted. |  |
| 6 | Line 49: Justify antibody assay when we collect data on antigen and Mf. It may not reflect the ongoing transmission. | The following text has been added at around line 396. “We recognise that antibody assay analysis for LF is still in its early stages, and results are challenging to interpret. However, we plan to include LF antibody testing in the MBA panel, as previous studies [29, 30, 34, 35] have shown that it can offer insights into disease epidemiology and help characterise pathogen transmission dynamics. Additionally, since parasite antigens are known to trigger an IgG response that is detectable over an extended period, serological data from younger children (who should not have been infected with LF in their lifetime) could signal ongoing transmission [36].” |  |
|  | ***Background*** |  |  |
| 7 | Line 64: what is the parasite species other than Wuchereria bancrofti prevalent in South Pacific countries? W.bancrofti is probably the only LF parasite prevalent in Niue [WHO. The PacELF way: towards the elimination of lymphatic filariasis from the Pacific, 1999-2005. Manila: WHO Western Pacific Region; 2006]. 8. Line 73: this article “Zeldenryk, L.M., Gray, M., Speare, R., Gordon, S., Melrose, W., 2011. The Emerging Story of Disability Associated with Lymphatic Filariasis: A Critical Review. PLOS Neglected Tropical Diseases 5, e1366.. <https://doi.org/10.1371/journal.pntd.0001366>” can be cited as reference for LF is the second leading cause of chronic disability. | Good pick-up, thank you. We have removed the word “predominant” from the sentence, so it now reads, “*W. bancrofti* is the parasite responsible for LF”.  The suggested reference has been added. |  |
| 8 | Line 76: Correct Ae. Cooki as Ae. cooki | This typo has been corrected. |  |
| 9 | Line 116: cite the reference of Carlingford [7] 11. ” | While Carlingford and colleagues are referred to in the sentence, we have added an in-text citation, as suggested. |  |
| 10 | Line 128-130: Suggest to rephrase the sentence “The survey was reported to have included 98.4% of a population, although nor whether this was for the entire population or a subset (e.g., those above a certain age) is unclear. | The sentence has been edited. Thank you for the helpful comment. |  |
| 11 | Line 140-142: Give prevalence data, if available instead of number of cases. [A relatively high number (n=13) of Ag-positive individuals were found in two locations (the villages of Tamakautonga and Hikutavake) that were distant to each other (i.e., not bordering each othe]. | Unfortunately, we do not have denominator data for these villages from that time. No change has been made. |  |
| 12 | Line 150: replace “were” by “where” | The typo has been corrected. |  |
| 13 | Line 156-157: add “in individuals older than 2 years” before [23] | This text has been added to the sentence. |  |
| 14 | Line 159-160: rephrase the sentence as “The seven individuals who tested positive in 2009 were re-tested for Ag and treated the positives until testing Ag-negative”. | The suggested text has been used. Thank you. |  |
|  | Method |  |  |
| 15 | Line 216: add “in all 14 villages” after diseases | This content has been added to the sentence. |  |
| 16 | Line 228: a column “total population” can be added before “Target sample size”. Add “in the age class 15-69 years after target sample size” | The table has been edited to include the reviewer’s suggestions. |  |
| 17 | Line 231-232: Migrants can also be excluded | The text around line 250 has been edited to read, “All Niue residents (regardless of citizenship) aged 15 to 69 are eligible to participate. The survey will include migrants to Niue, as previous hotspots of transmission were found in villages where migrant communities from neighbouring Pacific countries and territories congregate [7]. Migrant and mobile populations are also considered a high-risk group for LF transmission [25, 26]. Tourists and non-residents will not be included in the survey.” |  |
|  | ***Study implementation*** |  |  |
| 18 | Line 241: the target sample size for whole population (excluding below 15 years of age) LF survey can be mentioned. | This detail is stated in the preceding section titled “Sample size and study site selection”. Further, we do not think it sits well under the heading “Field Logistics.” No changes have been made. |  |
| 19 | Line 245 & 258: Consider providing a supplement table for “A standard STEPs survey questionnaire” | The questionnaire is in draft. We will include the finalised LF PVS integrated survey tool as a resource when we publish our results. |  |
| 20 | Line 253: preferably witness from either the community or health staff and witness by a survey team member can be avoided | The text has been edited to read, “a community member or Niue Department of Health staff member.” |  |
| 21 | Line 254: Assent form for individuals 15-17 years may be required | The text “an assent process” has been added to the sentence at around line 288. |  |
| 22 | Line 263: Whether GPS of the household will be recorded? | The text “including house geolocations” has been added to the sentence at around line 296. |  |
| 23 | Line 270: add “for LF survey” after blood collection tubes | The text “tubes for the LF survey” has been added to the sentence at around line 305. |  |
| 24 | Line 285: will the DBS samples be sufficient to carry out a panel of tests of other NTDs, VPDs, and VBDs? | Yes, we have adopted a protocol that has been tried and tested elsewhere, including studies led by our team in other Pacific Islands. |  |
|  | ***Discussion*** |  |  |
| 25 | Discussion brings out possible outcome of the study. However, discussion should focus on the timeline and how long these LF-PVS surveys if demonstrated feasible should be carried out. What changes are required when it is translated into a larger population? | In response, two changes to the discussion section have been made:   - new content has been added near the start of the discussion section (around line 359) that reads, “Post-validation surveillance for LF should be established once national validation is achieved; however, initiating PVS as early as possible after the second transmission assessment survey is beneficial. While further evidence is required to determine the optimal duration of PVS following validation, due to the lifespan of adult worms, the general sentiment is to maintain surveillance for at least 10 years.” and - new content has been edited at around line 411 to read, “While requiring adaptation to meet the local context, the protocol presented here provides a model others may apply. The results of our study (once implemented) will provide evidence for the feasibility of the approach and guidance for operationalisation. These insights will be valuable for those seeking to operationalise PVS in resource-constrained settings and meet the ambition to develop more efficient and integrated methods for surveillance and monitoring of NTDs set out in the WHO Neglected Tropical Diseases Roadmap 2030.” |  |
| 26 | Discussion may also indicate the frequency and duration of these surveys and interpretation of the results. The implications of showing Ag/Ab/Mf positives and any possible thresholds of each indicator may be discussed. | The following text has been added at around line 393, “With regards to LF PVS, an Ag positive result will indicate a person is infected with a live or recently deceased adult worm and indicate a potential source of transmission. A Mf positive result will confirm that the person is carrying breeding adult worms and is a transmission source. We recognise that antibody assay analysis for LF is still in its early stages, and results are challenging to interpret. However, we plan to include LF antibody testing in the MBA panel, as it can offer insights into epidemiology and help characterise pathogen transmission dynamics. Additionally, since parasite antigens are known to trigger an IgG response that is detectable over an extended period, serological status of younger children (who should not have been infected with LF in their lifetime) could signal ongoing transmission [34].The results of this survey will allow us to understand the status of LF in Niue. This information will be used to develop the next phase of activities and appropriate strategic responses. If no Ag-positive individuals are identified, we can be highly confident that LF has been eliminated as a public health problem in Niue; however, if there is low Ag prevalence or LF Ab are identified in individuals aged ≤20 years of age, our results will inform and direct an ongoing PVS strategy. If a high prevalence of Ag-positive or any Mf-positive individuals is identified, more intensive surveillance and targeted MDA will be considered.” |  |
