# Supplementary material for: Integrating post-validation surveillance of lymphatic filariasis with the WHO STEPwise approach to non-communicable disease risk factor surveillance in Niue, a study protocol
Source: PLoS One. 2025 Jan 16;20(1):e0315625. doi: 10.1371/journal.pone.0315625 (PMC11737670; doi:10.1371/journal.pone.0315625)
Supplement: Attachment — Submitted filename: Niue LF survey protocol_response to reviewers_R2.docx [file pone.0315625.s002.docx]

**Response to reviewers’ comments_R2**

**Ref number: PONE-D-24-36104**

**Title: Integrating post-validation surveillance of lymphatic filariasis with the WHO STEPwise approach to non-communicable disease risk factor surveillance in Niue, a study protocol**

| **Number** | **Comment** | **Response** | **Location in marked-up version of revised manuscript** |
| --- | --- | --- | --- |
| 1 | From the IRB-approved protocol, there appears to be (i) a standard STEPs household questionnaire and (ii) the addition of one question “To which countries have you travelled in the last 5 years?”, which is being added to this. **In view of this, please could you provide a copy of the STEPs household questionnaire or a link to the questionnaire, and add into the text details of the additional question** | We have edited the manuscript at around line 292-294 to address this comment.  The revised text reads: “The standard WHO STEPS survey tool ([www.who.int/publications/m/item/standard-steps-instrument](http://www.who.int/publications/m/item/standard-steps-instrument)) will be adapted to include one additional LF-related question: The additional question will be, “To which countries have you travelled in the last 5 years?” | Around lines 292-294. |
